# Combined Association of Tumoral PD-L1 Expression and Pretreatment Presence of Epstein-Barr Virus DNA With Risk Stratification and Prognosis of Patients With Nasopharyngeal Carcinoma

**DOI:** 10.3389/fonc.2021.791411

**Published:** 2022-01-18

**Authors:** Xiaoyu Li, Xingchen Peng, Sha Zhao, Hong Zhang, Yong Jiang, Fei Liu, Ping Ai

**Affiliations:** ^1^ Clinical Trial Center, National Medical Products Administration Key Laboratory for Clinical Research and Evaluation of Innovative Drugs, West China Hospital, Sichuan University, Sichuan, China; ^2^ Department of Biotherapy, Cancer Center, West China Hospital, Sichuan University, Chengdu, China; ^3^ Department of Pathology, West China Hospital, Sichuan University, Chengdu, China; ^4^ Department of Radiation Oncology and Department of Head & Neck Oncology, Cancer Center, West China Hospital, Sichuan University, Sichuan, China; ^5^ West China School of Medicine, Sichuan University, Sichuan, China

**Keywords:** PD-L1, EBV, nasopharyngeal carcinoma, metastasis, risk stratification

## Abstract

**Background:**

Little is known about whether the combination of tumor programmed death-ligand 1 (PD-L1) expression and pretreatment EBV DNA status can help stratify nasopharyngeal carcinoma (NPC) patients by risk of metastasis or predict prognosis.

**Methods:**

PD-L1 expression was assessed using immunohistochemical staining of 78 non-keratinizing NPC patients with clinical data. Survival outcomes and independent prognostic factors were identified.

**Results:**

Seventy-eight patients were included, high PD-L1 expression was observed in 25 of 43 patients (58%) with metastasis, while it was observed only in 7 of 35 patients (20%) without metastasis. Multivariate analyses showed that progression-free survival (PFS) was independently predicted by tumoral PD-L1 expression and pretreatment EBV DNA status. When combining, 93.75% patients with high PD-L1 and EBV infection developed distant metastasis, and those patients were associated with worse PFS.

**Conclusions:**

Both PD-L1 expression and pretreatment EBV DNA are closely related to metastasis and prognosis of NPC patients. Their combination can facilitate risk stratification and prognosis prediction, which may improve disease treatment and management.

## Introduction

Nasopharyngeal carcinoma (NPC), can be distinguished from other head and neck cancers based on its clinical presentation, pathological features, and epidemiology, as well as its close association with the Epstein-Barr virus (EBV) (1). Cases of NPC have been observed worldwide, and the reported number of new cases and the number of deaths in both males and females of NPC in 2020 were 133,354 and 80,008 worldwide. The age standardized incidence and mortality rates were both remarkedly higher in Southeastern Asia than in any other region (2–4). The main treatment modality for *early-stage* NPC is radiotherapy (5, 6). In patients with locally advanced NPC, treatment using a combination of radiotherapy and chemotherapy has increased the 5-year local control rate to 90% (7, 8). However, about 10-20% patients can experience recurrence or distant metastasis (9–12). The treatment options for recurrence and metastatic patients are limited, and the efficacy also needs further improvement (13). Thus, identifying effective markers for stratifying NPC patients by risk of metastasis may aid in their timely treatment and management.

NPC is a virus-driven malignancy (14, 15) that is characterized by EBV infection and the presence of immune cell infiltration around the cancer nests (16). Pretreatment and postradiotherapy plasma EBV DNA levels correlate with disease outcomes and long-term survival of patients with advanced NPC (17). Although pretreatment plasma EBV DNA has been suggested as a reliable biomarker for the diagnosis, risk stratification, and prognosis of NPC patients (17, 18), studies differ in the cut-off values that they apply (17, 19) and methods to quantify EBV DNA in plasma have not been standardized (19, 20). In addition, a randomized controlled trial analyzed plasma EBV DNA in NPC patients after chemoradiation to identify high-risk patients for adjuvant chemotherapy, results showed that adjuvant chemotherapy with cisplatin and gemcitabine did not improve relapse-free survival (21). This study indicated that the EBV DNA levels alone were unable to identify high-risk patients for adjuvant chemotherapy (21). There is an urgent need to identify efficient markers to help with risk stratification and development of treatment options for NPC patients.

Programmed death-ligand 1 (PD-L1), also known as B7-H1 or CD274, is a surface glycoprotein that induces T cell anergy or apoptosis by binding to its receptor, programmed cell death protein-1 (PD-1), on T lymphocytes (22, 23). PD-L1 is widely expressed on antigen-presenting and other immune cells, and is upregulated in tumor cells in a broad range of tumors, including NPC (24–28). The expression of PD-L1 is correlated with tumor grade or prognosis in several types of carcinomas (22, 23, 29), and patients with EBV-associated malignancies have high levels of PD-L1 (26, 30). PD-L1 has recently been reported as a prognostic indicator in NPC patients, but these findings remain controversial (31–34). Victor H. F. Lee and colleagues reported that patients with higher PD-L1 expression on tumors had longer locoregional failure-free survival and marginally longer PFS (31). Another study also demonstrated that overexpression of PD-L1 on tumor cells was associated with superior OS (34). However, several studies have shown that PD-L1 expression predicts poor prognosis for NPC patients. Yajuan Zhou et al. found that patients with a higher PD-L1 H-score could have a greater risk of death (33). A meta-analysis study including 1,315 patients revealed that PD-L1 overexpression was associated with a poor OS (32). These studies with inconsistent results indicated that PD-L1 alone may not be a perfect prognostic indicator of NPC.

EBV associated malignancies had distinct regulation mechanisms on the expression of PD-L1. In NPC, PD-L1 expression was higher in EBV-positive NPC cell lines than in EBV-negative cell lines, and that PD-L1 expression can be enhanced by the exogenous and endogenous induction of the latent membrane protein 1 (LMP1) and IFN-gamma pathways (35). EBV-encoded circular RNA also upregulated the PD-L1 expression in NPC by retinoic acid-inducible gene I (36). Therefore, a combination of PD-L1 expression and EBV infection status may be effective at identifying NPC patients at higher risk of metastasis or poor survival.

In this study, we examined the tumoral PD-L1 expression and pretreatment plasma EBV DNA status in a cohort of patients with non-keratinizing NPC to assess the combined effect of these two factors on risk stratification and prognosis prediction.

## Materials and Methods

### Patients

We included patients with a histologically confirmed diagnosis of non-keratinizing NPC and who received intensity-modulated radiation therapy at West China Hospital, Sichuan University (Chengdu, China) between January 2008 and August 2016. Biopsy specimens, a complete clinical history and follow-up data for at least three years were available for each included patient. We excluded patients with other malignant diseases, severe comorbidities, and those with Eastern Cooperative Oncology Group (ECOG) scores ≥ 2. This study was approved by the Institutional Review Board of West China Hospital of Sichuan University, and the need for written informed consent was waived by the Institutional Review Board.

### Data Collection

Medical records were reviewed for demographic information, including age, sex, smoking status, stage of disease, quantitative reverse transcription polymerase chain reaction (RT-PCR) data for plasma EBV DNA load at diagnosis, and survival outcomes. Disease stages were determined based on the American Joint Committee on Cancer tumor-node-metastasis staging (AJCC, 7th edition) (37). Progression-free survival (PFS) was defined as the time from diagnosis until progression, while overall survival (OS) was defined as the time from diagnosis until death due to any cause or the last follow-up.

### Quantitative RT-PCR

Peripheral venous blood (3mL) was obtained at the time of diagnosis (before treatment) and centrifuged at 1600 g for 15 min in tubes containing EDTA. Plasma EBV DNA was isolated and analyzed using a commercial kit (Sansure Biotech, Hunan, China) based on a quantitative RT-PCR assay (20, 38) that targets the BamHI-W fragment of the EBV genome. Any detectable amplification was treated as a positive result.

### Immunohistochemistry of PD-L1

Formalin-fixed, paraffin-embedded NPC tissues biopsied at diagnosis were retrieved from the pathology department. The sections were dewaxed, rehydrated through a graded alcohol series, placed in 95°C ethylene diamine tetra-acetic acid buffer (pH 8) for 40 min to retrieve antigens, then incubated in 3% H_2_O_2_ for 15 min at room temperature. The sections were stained overnight at 4°C with anti-PD-L1 primary antibody (clone SP142, ZSGB-Bio, Beijing, China), followed by incubation with secondary antibody for 45 min at 37°C and immunoreactions were visualized using the ChemMate EnVision+ detection kit (Peroxidase/Dab, Rabbit/Mouse, K5007, Dako, Glostrup, Denmark). Separate full slides containing NPC tissue of known PD-L1 status were used as positive and negative controls for PD-L1 staining. Tissue images were taken using a phase contrast microscope.

PD-L1 expression was semi-quantified in terms of the proportion of total cells that stained positive as well as the intensity of the positive stain by two experienced pathologists who were blinded to the clinical data. The optimal cut-off point for PD-L1 expression based on the percentage of positive tumor cells was determined based on the area under the receiver operating characteristic curve (ROC). Using the ROC value, the optimal cut-off point for tumoral PD-L1 expression was calculated as 45%. PD-L1 expression was dichotomized into two groups (high and low), when staining was observed on ≥ 45% of the tumor cells, it was classified as high expression.

### Statistical Analysis

Correlations between metastasis and PD-L1 expression or EBV status were assessed using the chi-squared test. Patients were stratified into four groups based on their tumor PD-L1 expression levels and pretreatment EBV infection status: (A) patients with low PD-L1 expression and no EBV infection, (B) patients with low PD-L1 expression and EBV infection, (C) patients with high PD-L1 expression and no EBV infection, or (D) patients with high PD-L1 expression and EBV infection. Factors affecting PFS and OS were assessed using the Kaplan-Meier method and univariate log-rank tests. Multivariate Cox regression was used to identify independent predictors of survival. Differences associated with two-sided p < 0.05 were considered significant. All statistical analyses were performed using SPSS 25.0 (Chicago, IL).

## Results

### Clinical Characteristics of Patients

In this study, we recruited a total of 78 patients with non-keratinizing NPC (63 men) with a median age of 44 and range of 22-65 years old. Follow-up data for at least 5 years were available for 94% of the patients; the median follow-up time was 60.4 months (range, 9.4-110.8 months). Of all patients, 43 (55%) had metastatic NPC (**Table 1**). The expression of PD-L1 was not significantly different between males and females or between smokers and non-smokers. In addition, the percentage of high PD-L1 expression was also not significantly different among different T stages and disease stages (**Table 1** *).*

**Table 1:** Association between PD-L1 expression and clinicopathological features of patients with NPC.

Variable	Category	n	(%)	Tumor PD-L1 expression		p value
				< 45% (n = 46)	≥ 45% (n = 32)	
**Age**						
	< 44	37	(47)	27 (59%)	14 (44%)	0.362
	≥ 44	41	(53)	19 (41%)	18 (56%)	
**Sex**						
	Female	15	(19)	9 (20%)	6 (19%)	1.00
	Male	63	(81)	37 (80%)	26 (81%)	
**Smoking status**						
	No	41	(53)	26 (57%)	15 (47%)	0.63
	Yes	37	(47)	20 (43%)	17 (53%)	
**T stage**						
	T1	17	(22)	9 (20%)	8 (25%)	0.936
	T2	17	(22)	10 (22%)	7 (22%)	
	T3	12	(15)	7 (15%)	5 (16%)	
	T4	32	(41)	20 (43%)	12 (38%)	
**N stage**						
	N0	5	(6)	5 (11%)	0 (0)	0.039
	N1	22	(28)	16 (35%)	6 (19%)	
	N2	36	(46)	16 (35%)	20 (63%)	
	N3	15	(19)	9 (20%)	6 (19%)	
**Disease stage**						
	I	1	(1)	1 (2%)	0 (0)	0.566
	II	14	(18)	10 (22%)	4 (13%)	
	III	21	(27)	11 (24%)	10 (31%)	
	IV	42	(54)	24 (52%)	18 (56%)	
**EBV status**						
	Negative	33	(42)	19 (41%)	14 (44%)	0.813
	Positive	42	(54)	26 (57%)	16 (50%)	
**Metastasis**						
	No	35	(45)	28 (61%)	7 (22%)	0.001
	Yes	43	(55)	18 (39%)	25 (78%)	

EBV, Epstein–Barr virus; PD-L1, Programmed death-ligand 1; NPC, nasopharyngeal carcinoma.

**Figure 1:**
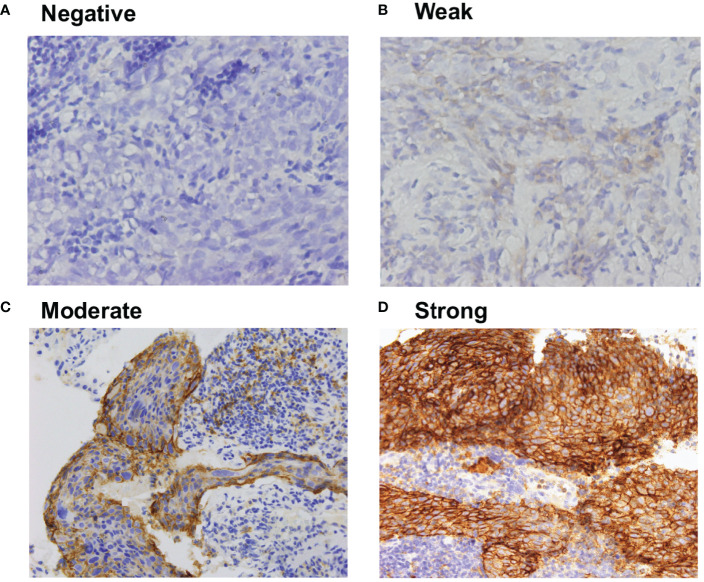
Representative immunohistochemistry of **(A)** negative, **(B)** weak, **(C)** moderate, and **(D)** strong PD-L1 staining in NPC tumor tissues. All the images are 40X magnification, The definition of negative was no staining, weak staining was yellow particles, moderate staining was yellow–brown, and strong staining was brown.

PD-L1 was detected in 66 patients (85%), primarily in the cytoplasm and membrane of tumor cells in a heterogeneous manner. Expression levels varied substantially across patients (**Figure 1**).

### High PD-L1 Expression and Positive EBV Status Are Associated With Metastasis and Worse Prognosis

**Figure 2:**
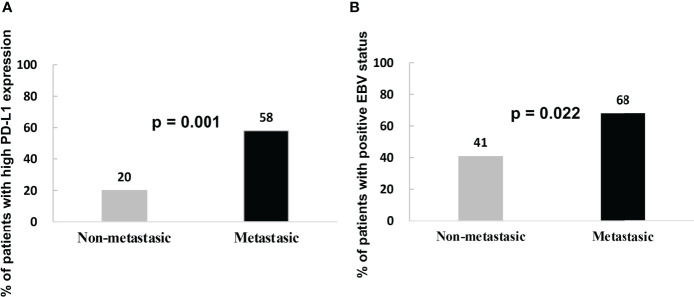
**(A)** Percentage of metastatic and non-metastatic NPC patients with high PD-L1 expression levels at diagnosis. **(B)** Percentage of metastatic and non-metastatic NPC patients who were EBV-positive at diagnosis.

**Figure 3:**
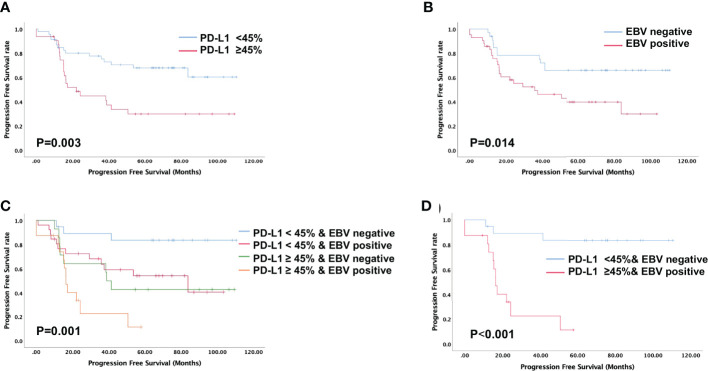
Evaluation of progression-free survival using the Kaplan-Meier survival analysis. **(A)** Patients with high PD-L1 expression is associated with shorter progression-free survival; **(B)** Patients with positive EBV status is associated with shorter progression-free survival; **(C, D)** Progression-free survival based on both PD-L1 expression levels and EBV status indicated that patients with high PD-L1 expression and positive EBV status is associated with shortest progression-free survival.

**Figure 4:**
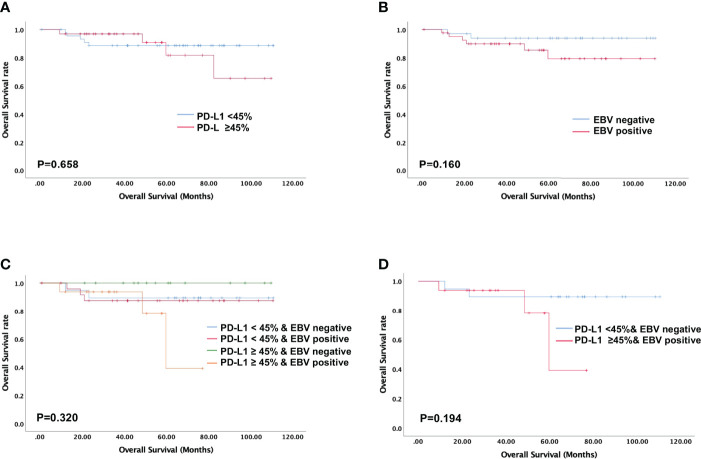
Evaluation of overall survival using the Kaplan-Meier survival analysis. No overall survival difference is noted in NPC patients stratified according to PD-L1 expression, EBV status, and their combination. **(A)** Overall survival based on tumoral PD-L1 expression alone; **(B)** Overall survival based on EBV status alone; **(C, D)** Overall survival based on PD-L1 expression levels and EBV status.

High PD-L1 expression was observed in 25 of 43 patients (58%) with metastatic NPC, while it was observed only in 7 of 35 patients (20%) without metastasis (p = 0.001, **Figure 2A**). This suggests that patients with high tumoral PD-L1 expression levels are more likely to develop metastatic NPC. In addition, Kaplan-Meier analysis showed that low PD-L1 expression was associated with better PFS (p = 0.003; **Figure 3A**) but not OS (p = 0.658; **Figure 4A**).

Similarly, serum EBV DNA was detectable at diagnosis in 28 of 41 patients (68%) with metastatic NPC, significantly higher than the frequency of 14 of 34 patients (41%) without metastasis (p = 0.022, **Figure 2B**). Undetectable EBV DNA at diagnosis was associated with significantly better PFS (p = 0.014, **Figure 3B**), but not OS (p = 0.160, **Figure 4B**).

### Tumor PD-L1 Expression Levels and EBV Status Are Independent Predictors of PFS

PFS showed a significant association with N stage, disease stage, EBV status, and PD-L1 expression (**Table 2**). Based on the univariate analysis, we found that high PD-L1 expression was associated with higher risk of disease progression (HR 2.66, 95%CI 1.36-5.19, p = 0.004), as was EBV positivity (HR 2.39, 95%CI 1.16-4.90, p = 0.014; **Table 2**).

**Table 2:** Univariate analysis of progression-free survival in patients with NPC.

Variable	Categories	HR (95%CI)	p value
**Age**	≥ 44 vs. < 44	1.776 (0.906-3.479)	0.216
**Sex**	Male vs. female	0.989 (0.433-2.258)	0.979
**Smoking status**	Yes vs. No	0.868 (0.431-1.747)	0.69
**T stage**	T3+T4 vs. T1+T2	1.095 (0.567-2.116)	0.786
**N stage**	N2+N3 vs. N0+N1	2.578 (1.172-5.670)	0.011
**Total stage**	III+IV vs. I+II	2.609 (0.921-7.394)	0.042
**EBV status**	Positive vs. Negative	2.387 (1.163-4.902)	0.014
**PD-L1 expression**	≥ 45% vs. < 45%	2.655 (1.359-5.185)	0.004

EBV, Epstein–Barr virus; HR, Hazard ratio; NPC, nasopharyngeal carcinoma; PD-L1, Programmed death-ligand 1.

Multivariate analysis identified three independent predictors of PFS in our cohort: N stage, high PD-L1 expression (HR 2.85, 95%CI 1.35-6.04, p = 0.006), and EBV-positive status (HR 2.90, 95%CI 1.41-5.97, p = 0.004) (**Table 3**). None of the clinical parameters showed a significant association with OS.

**Table 3:** Multivariate analysis of progression-free survival in patients with NPC.

Variable	Categories	Hazard ratio	95%CI	p value
**N stage**	N2+N3 vs. N0+N1	1.234	0.416-3.660	0.705
**Disease stage**	III+IV vs. I+II	2.103	0.514-8.609	0.301
**EBV status**	Positive vs. Negative	2.902	1.411-5.968	0.004
**PD-L1 expression**	≥ 45% vs. < 45%	2.853	1.347-6.042	0.006

EBV, Epstein–Barr virus; PD-L1, Programmed death-ligand 1; NPC, nasopharyngeal carcinoma.

### Association Between EBV Status and PD-L1 Expression in NPC Patients

Previous studies have reported that EBV infection could upregulate PD-L1 expression in NPC cell lines (35). Thus, we analyzed the association between EBV status and PD-L1 expression in all the patients. In the 33 patients with negative EBV status, 44% of patients had high PD-L1expression, and in the 42 patients with positive EBV status, the high PD-L1 expression rate was 50%. We did not observe a significant association between PD-L1 expression and EBV status by the chi-square test in our cohort (p=0.813, **Table 1**).

### Combination of EBV Status and PD-L1 Expression Levels Can Stratify Patients by Risk of Metastasis and Prognosis

In this study, we stratified the patients into four groups based on their EBV status and tumoral PD-L1 expression levels. When we compared the metastasis rates among these four groups, we found that patients with high PD-L1 expression levels and positive EBV status had the highest incidence of metastasis (93.75%), while the patients with low PD-L1 expression levels and negative EBV status had the lowest incidence of metastasis (26.3%). Patients with high PD-L1 expression and positive EBV status had the worst PFS among all four groups (p = 0.001, **Figures 3C, D**). The was no significant difference in overall survival of four groups (p=0.320, **Figures 4C, D**).

## Discussion

NPC is an EBV-associated malignancy with high metastatic potential (39), and metastasis is the most frequent reason for treatment failure (40). Therefore, it is important to identify a reliable and efficient marker to predict metastasis risk. In this study, we examined the joint role played by tumoral PD-L1 expression levels and EBV status in predicting metastasis risk and prognosis of NPC patients. Our findings reveal that patients exhibiting high PD-L1 expression and EBV-positive status at NPC diagnosis are more likely to experience metastasis and shorter PFS.

Several studies have reported correlations between plasma EBV DNA levels and the prognosis and treatment outcomes of NPC patients (17, 18). However, the stratifying power of EBV DNA levels on their own may be limited (21). Better results may be obtained by using a combination of two or more biomarkers, as shown for the combinations of tumor volume and EBV status, pretreatment EBV DNA levels and clinicopathological variables, or pretreatment EBV DNA levels and cervical node necrosis (41–43). However, none of these studies stratified patients based on treatment modality. With the current advances in immunotherapy, it is worth considering the addition of immunotherapy to chemotherapy, especially for patients at high risk of metastasis (44).

PD-L1, which is expressed on immune, antigen-presenting, and tumor cells, plays an important role in T cell tolerance and immune escape. This might be the reason why patients with higher PD-L1 expression had shorter PFS and correlates with metastasis in this cohort. The mechanisms of PD-L1 upregulation induced by EBV infection are complex. In NPC, PD-L1 expression was reported to be regulated by EBV-induced LMP1 and IFN-gamma pathways (35). Recently, Ge and colleagues found that EBV-encoded circular RNA CircBART2.2 upregulated the expression of PD-L1 in NPC and further inhibited the T-cell function (36). In other malignancies with positive EBV infection, IFN-gamma mediated signaling pathways, EBV microRNA, somatic gene mutations, and epigenetic modifications were also reported to regulate PD-L1 expression (45, 46). Therefore, PD-L1 may be an effective target for immunotherapy as well as a useful biomarker for predicting metastasis and survival. Many studies have investigated the role of PD-L1 expression and EBV in the prognosis and risk stratification of NPC, as well as other EBV-associated malignancies, but most of them did not combine tumor PD-L1 expression with EBV-DNA to further stratify NPC patients (30, 33, 47–51) Hu and colleagues stratified NPC patients into three groups based on the combination of EBV DNA load and PD-L1 expression on tumor infiltrating lymphocytes (TILs). The results indicated that a high log EBV-DNA load with low TIL PD-L1 was a poor prognostic factor for PFS (52).

The results of present study indicate that tumoral PD-L1 expression and pretreatment plasma EBV status act as independent prognostic indicators, and both of them are associated with metastasis in NPC patients. Our results show that patients with high tumoral PD-L1 expression levels and detectable EBV DNA have the highest incidence of metastasis and worst PFS, indicating that the combination of these two biomarkers can help identify high-risk patients. For those patients with higher risk for metastasis and worse survival stratified by the combination of PD-L1 and EBV status, more intensive anti-tumor treatment may necessary to reduce the incidence of metastasis and recurrence, further to improve patients’ survival. Adding anti-PD-L1/PD-1 immunotherapy to conventional treatment regimens for these high-risk patients may further prolong their PFS. Several PD-1 blocking antibodies have shown promising results in the treatment of recurrent and metastatic NPC failed on previous treatment (53–56). Recently, results of two-phase 3 clinical trials showed that anti-PD-1 antibodies plus gemcitabine-cisplatin chemotherapy as first-line treatment in advanced NPC patients had superior PFS compared to chemotherapy alone, and this regimen could be a new standard of care for metastatic or recurrent NPC (57, 58). This progress might help to improve the treatment efficacy of patients with high risk stratified by PD-L1 expression and positive EBV status.

To our knowledge, this is the first study to examine the combination of tumoral PD-L1 expression and pretreatment plasma EBV DNA status as biomarkers for risk stratification and prognosis in NPC. The EBV DNA status is routinely tested during the management of NPC, and PD-L1 expression can be evaluated using low-cost immunohistochemistry. Using these two indices together may provide good risk stratification without the need for complex nomograms. Although our study was able to identify biomarkers that can be used for risk stratification in NPC patients, our findings were limited by the small sample size of the cohort and by heterogeneity in PD-L1 expression within the same patient. Future studies should investigate these associations further and validate the relationship between metastasis and PD-L1 expression and EBV status using larger samples.

This study revealed that pretreatment plasma EBV status and tumoral PD-L1 expression levels are closely related to prognosis of NPC patients. Using the two biomarkers together may facilitate risk stratification and timely, effective treatment.

## Author’s Note

The work was presented at the 60th American Society for Radiation Oncology Annual Meeting, 2018.

## Data Availability Statement

The original contributions presented in the study are included in the article/supplementary material. Further inquiries can be directed to the corresponding author.

## Ethics Statement

The studies involving human participants were reviewed and approved by Institutional Review Board of West China Hospital of Sichuan University. Written informed consent for participation was not required for this study in accordance with the institutional requirements.

## Author Contributions

The corresponding author PA concepted and designed the study, edited and reviewed the manuscript. XL performed the experiment study, data analysis, statistical analysis, and manuscript preparation. XP worked on the clinical data acquisition, data analysis, statistical analysis and manuscript editing. SZ worked on the sample collection, data acquisition, data analysis, and manuscript editing. HZ worked on data acquisition. YJ worked on sample collection, experimental studies. FL searched the literature. All authors reviewed the manuscript and agreed with the submission.

## Funding

This study was supported by grants from the Department of Science and Technology of Sichuan Province (Grant No. 2020YFS0276), the Health Department of Sichuan Province (130087) and the Wu Jieping Medical Foundation (320.6750.13317).

## Conflict of Interest

The authors declare that the research was conducted in the absence of any commercial or financial relationships that could be construed as a potential conflict of interest.

## Publisher’s Note

All claims expressed in this article are solely those of the authors and do not necessarily represent those of their affiliated organizations, or those of the publisher, the editors and the reviewers. Any product that may be evaluated in this article, or claim that may be made by its manufacturer, is not guaranteed or endorsed by the publisher.

## Abbreviations

AJCC, American Joint Committee on Cancer; EBV, Epstein-Barr virus; ECOG, Eastern Cooperative Oncology Group; FDA, Food and Drug Administration; LMP1, latent membrane protein 1; NPC, nasopharyngeal carcinoma; OS, overall survival; PD-1, programmed death-1; PD-L1, programmed death-ligand 1; PFS, progression free survival; qRT-PCR, quantitative reverse transcription polymerase chain reaction; ROC, receiver operating characteristic curve; TILs, tumor-infiltration lymphocytes.
